# Towards approval of autonomous ship systems by their operational envelope

**DOI:** 10.1007/s00773-021-00815-z

**Published:** 2021-04-24

**Authors:** Ørnulf Jan Rødseth, Lars Andreas Lien Wennersberg, Håvard Nordahl

**Affiliations:** SINTEF Ocean, Postboks 4762 Torgard, 7465 Trondheim, Norway

**Keywords:** Autonomous ship systems, Operational envelope, Regulatory approval

## Abstract

Current guidelines for approval of autonomous ship systems are focused on the ships’ concrete operations and their geographic area. This is a natural consequence of the link between geography and the navigational complexity, but moving the ship to a new area or changing owners may require a costly re-approval. The automotive industry has introduced the Operational Design Domain (ODD) that can be used as a basis for approval. However, the ODD does not include the human control responsibilities, while most autonomous ship systems are expected to be dependent on sharing control responsibilities between humans and automation. We propose the definition of an operational envelope for autonomous ship systems that include the sharing of responsibilities between human and automation, and that is general enough to allow approval of autonomous ship systems in all geographic areas and operations that falls within the envelope. We also show how the operational envelope can be defined using a system modelling language, such as the unified modelling language (UML).

## Introduction

The concept of autonomous and uncrewed merchant ships has been around for some years now. The MUNIN project [1] started this wave in Europe from 2012, and other concept-ships were rapidly launched by different companies. DNV GL published its Re-Volt project in 2014 [2] and Rolls Royce published plans for the development of intelligent ships around the same time [3]. The world’s first test area was established in Trondheim, Norway in 2016 [4], and in 2017 Yara and Kongsberg published the first commercial contract to build an autonomous ship, the “Yara Birkeland” [5]. Today, there are a number of large national and regional research and development projects ongoing, both in Europe and Asia. The general expectation is that uncrewed and highly automated ships will be a significant factor in tomorrow’s sustainable ship transport system. Most large maritime nations are preparing to build and outfit such ships, as well as to make use of them in their internal and external trade.

IMO coined the term “Maritime Autonomous Surface Ship” (MASS) to describe this type of ship. It was agreed to add MASS to IMO’s Maritime Safety Committee’s (MSC) agenda and to initiate a regulatory scoping exercise in 2017 [6]. The scoping exercise started in 2018 with the provisional definition of MASS as follows: “For the purpose of the regulatory scoping exercise, Maritime Autonomous Surface Ship (MASS) is defined as a ship which, to a varying degree, can operate independent of human interaction” [7]. The scoping exercise will end at MSC 103 in 2021, after some delay due to COVID-19. Any concrete work on regulations will not start before this exercise is finished and it is expected to take many years before new international rules can be ready. In the interim period, approval of new autonomous ships will need to be done nationally or regionally and is expected to be based on the IMO Circular MSC.1/Circ.1455 [8] on “Approval of Alternatives and Equivalents.” Norway, as the first flag state, has published a national circular based on the IMO guidelines that gives guidelines for approval of MASS [9].

The Norwegian circular, as well as several of the classification societies’ guidelines, require a risk analysis based on the ship’s intended operations in a specific geographic area. This makes sense as the concept of MASS is very new and there is not much knowledge about how to design or approve such ships. However, it also limits the approval to the specific operations and the geographic area, and makes it difficult to move the ship to a new operation or to transfer it to another operator. To address this problem while still catering for the novelty of the concept, we propose to introduce the “operational envelope” as a generalization of the operational scenario. The operational envelope is an extension of the idea of the Operational Design Domain (ODD) that has been proposed used for cars [10] and which was also initially proposed for autonomous ships [11]. The term ODD is also used in ClassNK guidelines [12], but in a similar meaning to the operational envelope as discussed here.

Section 2 of this paper contains a review of the regulatory framework for the approval of MASS, and investigates how the proposed operational envelope concept fits into this picture. Section 3 will provide some definitions related to ship autonomy, including a proposal for the classification of ship autonomy degrees. Section 4 will introduce our definition of the operational envelope. Section 5 will give a more detailed description of the operational envelope concept, how it is linked to UML use cases, and how it can be used in a re-approval process. Section 6 will give some simple examples of how UML also can be used in other part of the control system design process. Section 7 is a summary of the proposals and conclusions made in the paper.

## The regulatory framework and its link to operational scenarios

It is important to recognize that virtually all autonomous ships must be considered as autonomous ship systems. Larger ships will normally require supervision and assistance from a remote control centre (RCC), they will often rely on shore-based automation, e.g. automated mooring, battery charging or cargo handling, and they will be dependent on communication systems, external position reference systems and other infrastructure. Without the total system, the ship itself would be virtually useless. Thus, MASS could perhaps better be an abbreviation for “Maritime Autonomous Ship System” [13].

This is also obvious from flag state and classification society guidelines for design and approval of MASS. Most refer to design and approval of the ship system rather than the ship itself. This unfortunately also requires a heavy emphasis on the specific operations and the geographic area that the ship is intended to operate in, which creates a dependency between the operational scenario and the ship’s license to operate.

Several classification societies, as well as the Norwegian Maritime Authority (NMA), have issued guidelines for the approval of autonomous ships. Their approach is largely based on the IMO circular MSC.1/Circ.1455 [8]. The circular defines a design and approval process for “alternatives and equivalents” where a “Preliminary design” and “Final design” description are central. The circular does not specifically target autonomous ship technology, but “ship functions, systems or components that either directly or indirectly proposes alternative ways of compliance with prevailing regulations”.

A common theme in the guidelines is that the operational scenario is a central part of the approval basis. Examples of guideline concepts which in various ways include the operational scenarios are: Operational Design Domain [12], Concept of Operations (ConOps) [9, 14–16], and Operational Limitations [17]. We have studied these guidelines to investigate whether the operational envelope can be used to define the operational scenario. An overview of what types of information that are required by the different guidelines is given in Table 1. Two abbreviations are used in the table: Env. (environmental factors) and Resp. (human/automation responsibility).

IMO and the European Union have also issued guidelines for trials of MASS [18, 19], however these guidelines do not address the approval of MASS, so these have not been included in our study.

**Table 1:** Approval basis information versus operational envelope

Guideline	Env.	System	Resp.	Functions	Area
NMA	X		X	X	X
DNV GL	X		X	X	X
BV	(X)	(X)	X	X	(X)
ABS	X		X	X	X
ClassNK	X	X	X	X	X
LR	X			X	X

The NMA circular [9] requires that a ConOps is submitted. The ConOps shall contain a description of the environmental conditions, the operational area, the operations that the ship shall execute, the functions, and the responsibility division between humans and automation. The circular does not discuss documentation of how system conditions are considered in the function design. Notably, the circular states that the ConOps is to be updated if the ship’s operational area is changed.

The DNV GL guideline [14] requires that a ConOps document is submitted as part of the Preliminary Design. The ConOps includes the environment conditions, the operational area(s), the functions and the responsibility division. It seems that they do not discuss documentation of how system conditions are considered in the function design. They do however discuss that own vessel’s condition is a part of the situational awareness. They also propose that sophisticated diagnostic functions such as condition and health monitoring is included, and that unexpected conditions are detected and alarmed.

The BV guideline requires several documents to be submitted [17]. One of the required documents is an operational limitations document. The document is defined as “The operational limitations of a ship are parameters to which the crew or operators must refer for the monitoring and control of the ship”, and “It is the designer, shipyard, manufacturer and/or shipowner responsibility to specify these limitations to define the conditions under which the ship is to be operated”. The document should contain descriptions of all functions and their degree of automation, degree of direct and remote control, and navigational notation. A separate Automation systems document shall be submitted for detailed specification of all functions and also to clearly define the division of roles and responsibilities between crew, automation and RCC. While traffic conditions are explicitly identified as a part of the operational limitations document, environmental conditions such as weather and geography or system conditions are not. It is however likely that they are to be included considering that the operational limitations are to be specified: “...in order to define the conditions under which the ship is to be operated”. A separate document for interactions with other ships is required, and the environmental conditions are also indirectly a part of the operational limitations document, as it is to include the navigation notation. The navigation notation is one of the following: unrestricted navigation, summer zone, tropical zone, coastal area or sheltered area.

Notably, the BV guideline does not require that the specific operational area is a part of the approval basis documentation, though they state that “In special cases, the designation of the geographic area and/or the most unfavourable sea conditions considered may be added to the navigation notations”.

The ABS guideline [15] describes a ConOps document which includes environmental conditions, operational area, and a description of functions and the related division of responsibility. It does not discuss documentation of how system conditions are considered in the function design. Notably, they require that an operational envelope is part of the ConOps. However, they list geographical operational area as an example of the content in the operational envelope, which is in contrast to our definition that is independent of the geographical operational area.

The ClassNK guideline [12] lists documents which are to be submitted for type approval of autonomous operating systems and for plan approval of individual ships. One of these documents is the ODD document. The ODD document defines the design range that the Autonomous Operating System and Remote Operating System can work properly under. They specifically mention environmental conditions, traffic conditions and geographical operational area as examples. However, given their general definition of ODD it can be assumed that the system conditions also will have to be described. Documents describing each function, how they are implemented, and how responsibility is divided between humans and automation are also required.

The Lloyd’s Register Code for Unmanned Marine Systems [16] requires a ConOps document which includes environmental conditions, service area (geographical operational area), and a description of functions. There is no discussion on documentation of how system conditions are considered in the function design. There is also no mention of responsibility division between human and automation.

Several of the guidelines do not explicitly require documentation of how system conditions are considered in the function design. However, considering that the system conditions (e.g. full sensor availability as opposed to loss of one sensor) will have a significant impact on the functionality, we believe that system conditions must be a part of the approval basis. The other main elements of the operational envelope, as shown in Table 1, are parts of the required documentation, with the exception of the responsibility division which is not discussed by Lloyd’s Register.

All but one of the studied guidelines requires the geographical operational area to be a part of the approval basis documentation. BV, which does not require the operational area to be included, opens up for it being amended.

Although the definitions and details on what information that is to be included in the approval basis varies, all these guidelines points to the same thing; the documentation that is the basis for approval must include descriptions of the MASS (system) design, the intended operations and the environment it is intended to operate in. These elements are also included in the operational envelope. Thus, the current regulatory framework for approval of autonomous ship systems provides important arguments for the use of the operational envelope:

- The operational envelope could serve as a standardised format capturing the functions and responsibility division under the various operational scenarios that the system is designed for.
- It could also be used to provide more formal representation of the Preliminary and Final design documents [20].
- Finally, the operational envelope can provide a means to do this without limiting the approval to a specific geographical operational area.

## Characterisation of ship autonomy

The definition of autonomy, and the difference between automatic and autonomous, is a much debated theme in the literature. The issue is also discussed in [13] and there we propose the following definition of automatic: “pertaining to a process or equipment that, under specified conditions, can function without human intervention.” This definition is based on IEC 60050-351 [21], with the addition of “can” to emphasize that human attention may be required, even in highly automated systems, and that the effected human attention often is based on the human’s own assessment of the situation. In the same paper, a slightly paraphrased version of a proposed definition of autonomous is: “in the context of ships, autonomy means that one or more of a ship system’s processes or equipment, under certain conditions, is designed and verified to be controlled by automation, without human assistance”.

The proposed definition of autonomy is basically the same as the “weak autonomy” definition in [22], and the arguments for using this particular definition are [13]:

1. The term autonomy is already in common use, e.g. in the term MASS, and it is better to give it a clear and useful definition than trying to depreciate it as, e.g. proposed by SAE [10].
2. Other “stronger” [22] definitions of autonomy are difficult to quantify, i.e. including learning or perception as prerequisites for autonomy, requires that these concepts are defined so that we can differentiate between, e.g. a sensor-based adaptive controller and “real” autonomous control.
3. The requirement that autonomy is able to function without a human in the loop, captures the main new regulatory challenge: approving automatic control of certain ship processes that completely remove the humans from the control functions for shorter or longer periods.

The critical difference between the proposed definitions of automatic and autonomous is that autonomy requires automation, but appears only when the system is designed and verified to operate without human assistance. In addition, the proposed definition of autonomy uses the term “under certain conditions”. This means that there normally is a temporal aspect to autonomy, and that the system will need to go from automatic to human-controlled operation, when the conditions are no longer met.

The temporal aspect allows us to characterise autonomy by two factors, $T_{\text {MR}}$ and $T_{\text {DL}}$, where $T_{\text {MR}}$ is a measure of the degree of human control and $T_{\text {DL}}$ of the degree of automation. $T_{\text {MR}}$ is the maximum response time and in a given operational scenario, it is defined as the maximum time the operator will need to reach the control position, gain situational awareness and be ready to perform actions to maintain safety. $T_{\text {DL}}$ is the response deadline and it is defined as the minimum time that the automation can maintain safe operation of the relevant process or equipment, in the same specific operational scenario. We use these two factors to define four degrees of automation and four degrees of human control, as shown in Table 2.

**Table 2:** Degrees of automation and human control in MASS

Degree	Of automation	Of human control
0	Low ($T_{\text {DL}} = 0$)	None ($T_{\text {MR}} = \infty$)
1	Partial ($T_{\text {DL}} > 0$)	Available ($T_{\text {MR}} > \sim 20 min$)
2	Constrained ($T_{\text {DL}} > t$)	Discontinuous ($T_{\text {MR}} >\sim 1min$)
3	Full ($T_{\text {DL}} = \infty$)	Continuous ($T_{\text {MR}} \sim 0$)

The low and full degrees of automation, respectively require continuous human attention and allow fully unmanned operation. The partial degree of automation refers to a situation where the automation can maintain control for some time, but where it is not possible to calculate this time by the automation system. The operator must continuously use own judgement if he or she wants to leave the control position. Constrained automation means that the automation system can calculate the response deadline and the automation system will be able to alert the operator at least $T_{\text {DL}}$ before it is necessary to take control. This defines a requirement for safe operation when $T_{\text {DL}} > T_{\text {MR}}$.

The degree of human control is directly related to the length of $T_{\text {MR}}$. The times given in Table 2 are rough indications, and the actual values will depend on relevant operational procedures. The values shown reflect situations such as when the crew is sleeping and must be mustered to the control position (e.g., 20 min), or when the crew is awake but performing some other task and must be alerted to change tasks (e.g., 1 min).

**Fig. 1:**
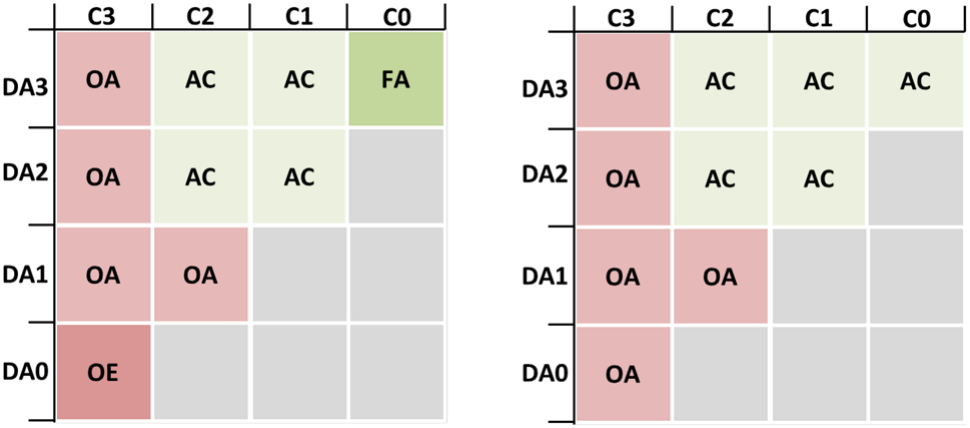
Possible combined degrees of control and automation

We can characterise autonomy by the different combinations of degrees of human control and automation as shown in Fig. 1. Here, DAn represents degree of automation *n* and Cm represents degree of control *m*. Note that human control can be exercised from a remote control centre or from the ship, so *m* is the maximum degree of control exercised from any of these positions.

The left-hand side of the figure shows all the relevant combinations of degrees of control and automation, and the resulting characterisation of autonomy:

- *FA*—*full autonomy* No operator is at the control position, and automation is able to handle all expected events in this state.
- *AC*—*autonomous control* The operator is away from the control position for a known period ($T_{\text {MR}}$) and can, when necessary be alerted by the automation with sufficient time to get back ( $T_{\text {DL}} > T_{\text {MR}}$).
- *OA*—*operator assisted* The operator is near the control position at all times. The operator can leave the control position for shorter periods but need to exercise own judgement as to how long he or she can be away.
- *OE*—*operator exclusive* The operator must be at the control position at all times.

The grey unlabeled areas in the lower right corners represents impossible states, i.e. too low degree of automation to handle the operator absence. One should note that most autonomous ship systems will move between different areas in this matrix, dependent on the external and internal conditions. An example is that the bridge may be unmanned during night and in good weather (e.g., DA2, C1), while it is fully manned during port operations (e.g., DA0, C3). The right-hand side figure represents a simplification of the four cases into two cases:

- OE and OA can often be merged to OA, as OE just means that the operator’s judgement should be to stay at the controls at all times ( $T_{\text {DL}} = 0$).
- Unless the ship is truly without human control, FA can be merged into AC as FA just means that $T_{\text {DL}}$ is very long when the system is in this state.

It is expected that most autonomous ship systems will operate with continuous supervision from an RCC. In this case, it may be more convenient to use the right-hand side classification to simplify the definition of responsibilities for the human operators.

The characterisation of ship autonomy by temporal properties is a different approach compared to what is found in the guidelines discussed in Sect. 2. We believe that this is an useful approach to characterisation as it clearly defines the responsibilities of the automation system versus humans, in terms of response times. From an approval point of view, this may be a clearer criterium than autonomy levels based on e.g., how far into the “human information processing pipeline” the automation operates [23].

## The operational envelope

There are opposing views on how humans should be involved in the control of autonomous vehicles. The automotive industry focuses on development of autonomous driving capabilities completely without humans in the loop, whereas the maritime industry largely expects that the responsibilities for control and monitoring of autonomous ship systems must be shared between humans and automation.

The different views stem from some important features that distinguish road vehicles from ships. Ships, especially large ones, have a much higher cost and damage potential compared to road vehicles. This makes it more cost-effective to invest in remote control centers with personnel that can supervise autonomous ship operations and also intervene in complex and potentially dangerous situations. Another feature is that most merchant ships move slowly and have more space to use for manoeuvring, compared to road vehicles. This makes it easier to rely on the operator being able to gain sufficient situational awareness and to intervene when the automation is incapable of maintaining control.

A third feature that favour human supervision for ships, is inherent in the automatic control algorithms. Most control systems rely on models of the physical systems that are to be controlled. Physical factors such as geographic obstacles, weather and visibility are possible to model. However, it is generally impossible to know what a human at the helm of another ship will do in a complex situation. The system may predict a probable outcome, but the risk in basing decisions on this prediction may still be unacceptably high. The navigation problem may be overcome by adopting more automation-friendly regulations or by implementing new information exchanges between the ships [24], but until such regulations and standards are available, it will often be necessary to use a human operator to limit risk and liability.

The higher involvement of humans requires that we consider the responsibilities of and the interface between the automation and the human operators in an autonomous ship system. For this reason, we propose to extend the operational design domain (ODD) used for road vehicles to an operational envelope for autonomous ship systems, that also includes the human’s responsibilities.

**Fig. 2:**
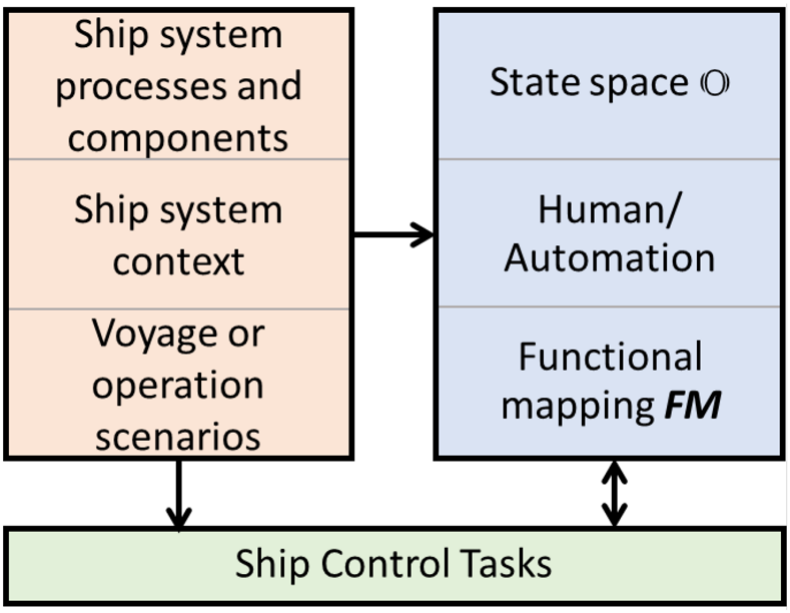
Information related to the operational envelope

The operational envelope ***O*** is defined as “The specific conditions and scenarios under which a given autonomous ship system is designed to function” [25]. The conditions shall include geographic or fairway conditions, environmental conditions, traffic conditions, as well as any other factors that have a significant impact on the operation of the autonomous ship system. Furthermore, the operational envelope shall cover all voyage and operation phases, relevant autonomous ship system processes and the division of responsibility between human and automatic control. Some of these relationships are illustrated in Fig. 2. The left boxes represent the mission or voyage scenario and context as well as ship capabilities, the right boxes represent the operational envelope, and the bottom box the ship control tasks (SCT). SCT is defined as “all of the process control tasks, implemented by automation and/or humans, that are required to sustainably operate the autonomous ship system within its operational envelope” [25].

In [25], ship system processes are schematically broken down into operations, functions, tasks and sub-tasks. How this sub-division is done for a real ship system, will depend on the system, the intended operation, and the designer. In the following, we will use the term function and function mapping to refer to a set of ship control tasks that together control one such sub-division of the ship system processes. As an example, a function like navigation in sheltered water could be divided into three tasks: track and speed control, object detection and classification, and obstacle avoidance.

We can categorize the elements of the operational envelope along two main axes. The first defines the state space ${\mathbb {O}}$ of the operational envelope. The state space is defined by the autonomous ship systems’ external and internal states, as defined by the left boxes in Fig. 2. The external states are the environmental conditions that the autonomous ship system is subject to. The internal states are the technical conditions of the autonomous ship system. Some of the state variables are operational constraints, e.g. daylight operation only or requirements for automatic mooring systems, and will not be used to control the ship systems processes. However, such constraints are also included in the operational envelope and in ${\mathbb {O}}$.

The second axis of the operational envelope is the function mapping ***FM***. It links SCT to a sub-space ${\mathbb {M}}_{jk}$ of ${\mathbb {O}}$ and allocates the responsibilities for the execution of these tasks to human operators and/or automation. The index *j* represents SCT, and the index *k* the state-space component. Index *k* will often be linked to a use case index *i*, but not necessarily. See Fig. 5 for examples of a use case split over two functions as well as functions that covers more than one use case.

Some parameters that can be used to define the operational envelope state space and the function mapping are given as examples in Table 3, where each row represents one example of environment and system conditions, function and responsibility mapping.

**Table 3:** Elements of the operational envelope

State space ${\mathbb {O}}$		Function mapping ***FM***	
Environment	System	Function	Resp.
Traffic density	Sensors	Navigation	Both
Wind	Engine state	Energy prod.	Automation
Temperature	Ship stability	Cargo handling	Human

The following equations will give some simple requirements to and relationships between use cases, function mappings and the state spaces. Refer also to Fig. 5 and associated description in Sect. 5 for examples.

1 $$\begin{aligned}&{\mathbb {O}} = \bigcup _{i} {\mathbb {U}}_i \end{aligned}$$

2 $$\begin{aligned}&{\mathbb {O}} \cong {\mathbb {M}}_j = \bigcup _{k} {\mathbb {M}}_{jk} \end{aligned}$$

3 $$\begin{aligned}&\forall j : \bigcap _{k} {\mathbb {M}}_{jk} = \emptyset \end{aligned}$$

4 $$\begin{aligned}&\forall \mathbf{c} \in {\mathbb {O}}, \forall j, \exists k : \mathbf{c} \in {\mathbb {M}}_{jk} \end{aligned}$$

The operational envelope will be defined from a number of UML use cases, indexed with *i*, that need to cover all important aspects of the ship’s intended mission or voyage. The state space for the operational envelope is the union of state spaces for the use cases (Eq. 1).

The functional mapping state space for a specific function *j*, will be the union of all functional mappings over all relevant state space components (Eq. 2). This must be congruent with ${\mathbb {O}}$ to ensure that there is a relevant SCT for all states in the operational envelope. Some mappings may refer to an “inactive function”, as e.g. cargo handling does in Fig. 5.

To ensure an unambiguous mapping from any state in ${\mathbb {O}}$ to the correct function, it is necessary for the designer to ensure that all functional mappings *k* for a given function *j* are disjunct from each other (Eq. 3).

Finally, there must always be a mapping from any state vector **c** in ${\mathbb {O}}$ to a valid function (Eq. 4). This corresponds to the congruence with ${\mathbb {O}}$ in Eq. 2.

**Fig. 3:**
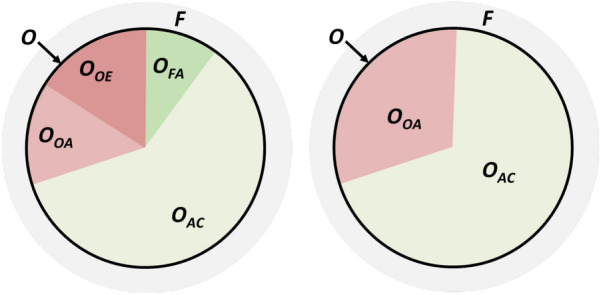
Representation of the operational envelope for autonomous ship systems

Figure 1 showed how the degree of control and degree of automation can be combined to define different degrees of autonomy. This concept can be transferred to the operational envelope as illustrated in Fig. 3.

***O*** in Fig. 3 shows the boundary between where automation and human can control the ship system processes and where “fail-to-safe” procedures need to be activated. For ship systems, the latter is called fallback to “Minimum Risk Condition” (MRC), as it may be impossible to define a fully safe condition, e.g. for a ship at sea with a stopped engine. The set of MRCs is called the fallback space ***F*** and is outside the operational envelope.

For a state ***c*** in ${\mathbb {O}}$, there will be *j* different functional mappings ${\mathbb {M}}_j$, as shown in Eq. 2. Each of the *j* corresponding functions will generally have a different $T_{\text {DL}}$ for ***c***. This means that the overall degree of autonomy of the ship system, as indicated in Fig. 3 will depend on the minimum $T_{\text {DL}}$ of the different functions. With *FDL* being a function to calculate $T_{\text {DL}}$ for a functional mapping ${\mathbb {M}}_j$ and a state ***c***, this can be expressed as:

5 $$\begin{aligned} \forall \mathbf{c} \in {\mathbb {O}}: T_{\text {DL}} = \min _j {\text {FDL}}({\mathbb {M}}_j, \mathbf{c} ) \end{aligned}$$

Defining a complete set of use cases and what functions to be described in each use case, is critical for the approval process. The set of use cases and functions must cover all critical aspects of the ship’s operation. To aid in this, a template process breakdown as well as template voyage or mission phases have been proposed in [25]. Ensuring the completeness of the operational envelope is still being researched, but we believe that a standardized and structured approach, e.g. based on UML, will help significantly to achieve this.

## The operational envelope as a generalization of the operational scenario

Case by case approval of autonomous ships as discussed in Sect. 2, requires that large parts of the approval process may need to be redone in the event of a transfer of the ship to another operator or another geographic area.

**Fig. 4:**
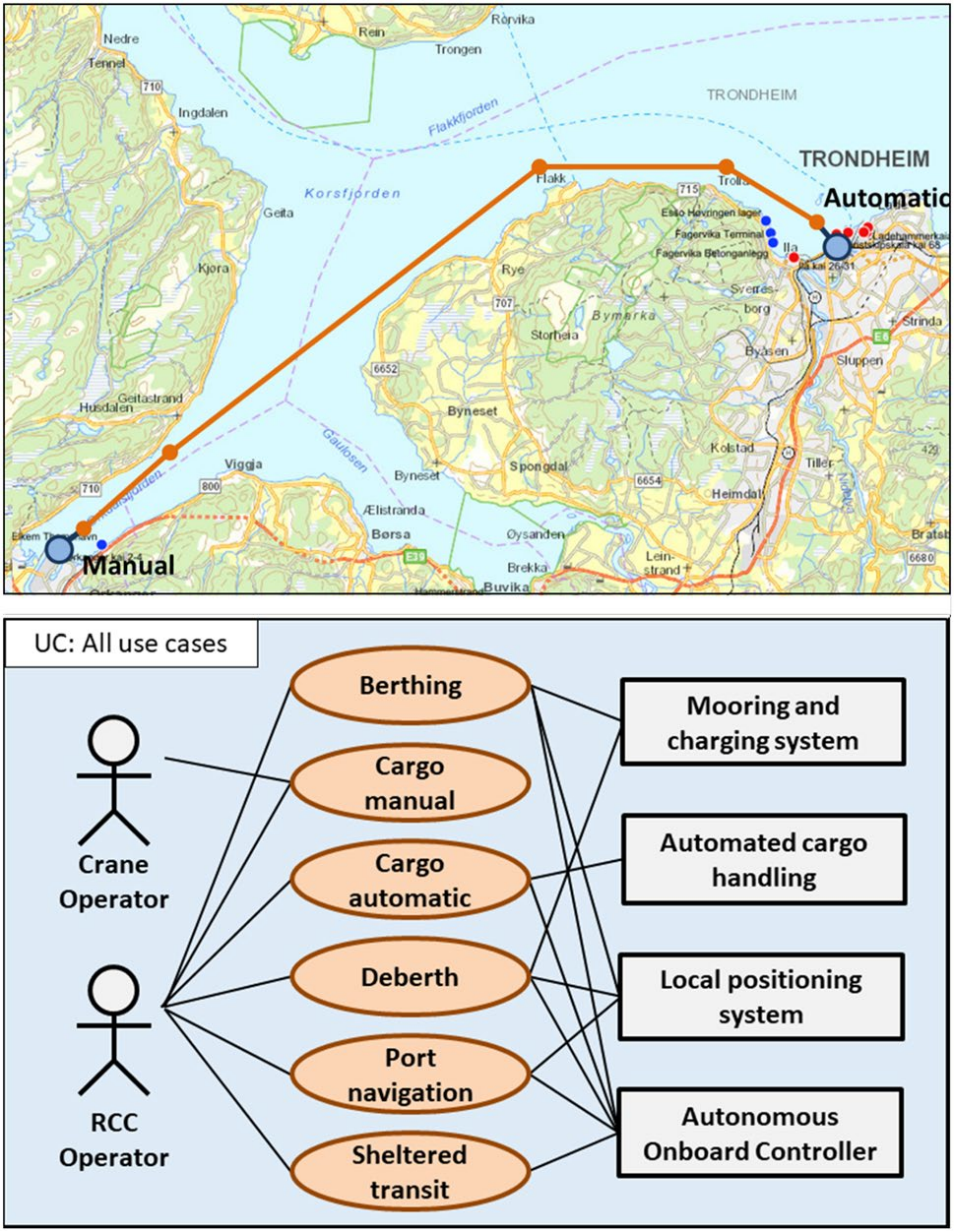
Converting an operational scenario to use cases (map from https://kystinfo.no/)

To enable a more flexible approval process, we need a more generalized description of the ship’s operations. The operational envelope is based on state-space descriptions and can be used to do this generalization. This paper proposes to use UML mechanisms to transfer operational scenarios to state spaces. The initial process is illustrated in Fig. 4 where an operational scenario on the top has been converted to more general UML use cases at the bottom [25].

The use cases are defined from the operational scenario by breaking the scenario down into mission phases and then generalize all mission phases to a smaller set of use cases. This is illustrated in the upper part of Fig. 4, where one autonomous ship voyage from Orkanger to Trondheim has been broken down into 10 different legs and port operations. A return trip will add another 10 mission phases to the total. In the lower part of the figure, the 20 operations are generalized into six use cases. These are defined over state spaces ${\mathbb {U}}_1$ to ${\mathbb {U}}_6$. The state variables will be, e.g. under keel clearance, traffic density, cargo volumes, ship speeds, ship stability for loading and discharge etc. Some of the state variables will be constraints, e.g. a maximum wave height of two meters or operation in daylight only.

**Fig. 5:**
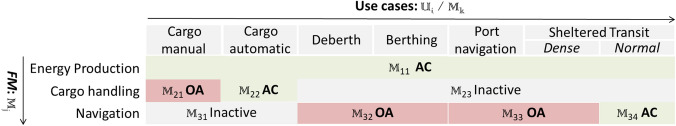
Breaking use cases down to function mappings

The use cases must be broken down into a functional mapping, and this is illustrated in Fig. 5. This shows a breakdown to three example functions: energy production, cargo handling, and navigation. More will normally be needed for a real design. The example creates eight functional mappings ${\mathbb {M}}_{11}$ to ${\mathbb {M}}_{34}$. Each are marked as autonomous control (AC), operator-assisted control (OA), or inactive. The functional mappings do not map one to one to use cases, as ${\mathbb {M}}_{11}$ and ${\mathbb {M}}_{33}$ show. The first cover all use cases while the latter splits a use case, as we assume that navigation in port and in dense traffic is the same operator-controlled function. Similarly, berthing and deberthing is assumed to be the same operator-controlled function, that also requires controlling the mooring and charging systems.

This shows how an operational scenario, consisting of 20 individual operations can be reduced to six use cases and eight functional mappings, including allocation of responsibilities between automation and humans. This will represent the operational envelope for the autonomous ship system. Section 6 will show some examples of how this can be further developed into more detailed UML specifications.

Changing the ship’s operational scenario should now be straight forwards as long as the new scenario has constraints and variables that are covered by the existing operational envelope. The verification process is illustrated by Eqs. 6 and 7. The two operational scenario state spaces are defined by the respective use case state spaces (Eq. 6). If it can be shown that for each of the new ${\mathbb {U}}_{2j}$ that it is a sub-set of an existing use case state space ${\mathbb {U}}_{1i}$, then the new operational state space ${\mathbb {O}}_2$ must be a sub-set of the original ${\mathbb {O}}_1$ (7). This also means that there will be valid and verified function mapping from all state vectors in ${\mathbb {O}}_2$ to the original function mapping ***FM***.

6 $$\begin{aligned}&{\mathbb {O}}_1 = \bigcup _{i} {\mathbb {U}}_{1i}, \;\; {\mathbb {O}}_2 = \bigcup _{j} {\mathbb {U}}_{2j} \end{aligned}$$

7 $$\begin{aligned}&\forall j, \exists i : {\mathbb {U}}_{2j} \subseteq {\mathbb {U}}_{1i} \Rightarrow {\mathbb {O}}_2 \subseteq {\mathbb {O}}_1 \end{aligned}$$

This means that the re-approval for operation, e.g. in a new geographic area, in principle can be reduced to proving that the new use cases are sub-sets of the ones that form the approval basis for the autonomous ship system. In practical terms, this proof can be based on the allowed range of the different state variables and constraints.

## Examples of UML used in system design

**Fig. 6:**
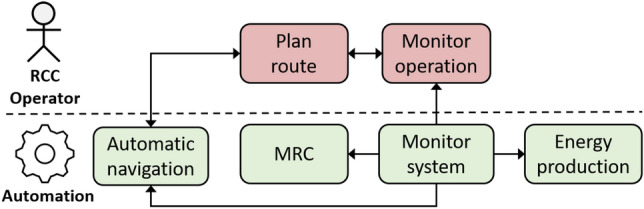
Activity diagram for normal traffic conditions in sheltered transit use-case

**Fig. 7:**
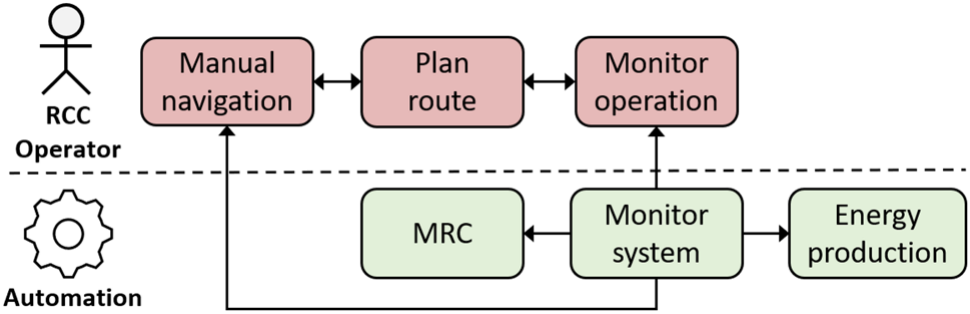
Activity diagram for dense traffic conditions in sheltered transit use-case

**Fig. 8:**
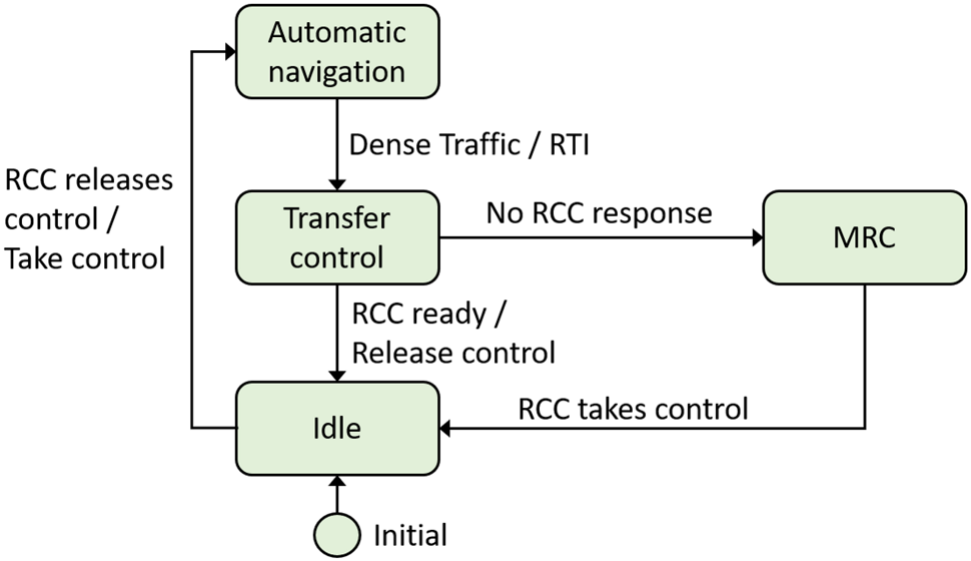
State diagram for automation

The bottom part of Fig. 4 uses graphic conventions from the Unified Modelling Language (UML) tool set [26]. This particular diagram shows UML use cases, including some important actors involved in the use cases. As has been shown in the previous section, this can be used as the basis for definition of the operational envelope and as part of the approval basis for the autonomous ship system. The use of UML constructs have an additional benefit in that UML can be used in the design and specification of the control systems. This section will give a few simplified examples of how this can be done through other types of UML tools. Some examples of relevant UML tools are listed in Table 4. Figures 6, 7, and 8 show how the two middle tools in the table can be used to describe additional details in the use case Sheltered transit from Fig. 4. This is based on a case that is more extensively described in [27].

**Table 4:** UML components used to describe the operational envelope

UML diagram type	Describes
Use case	Operations and conditions
Activity diagram	Interactions, responsibility
State machine	Detailed functions
Sequence diagram	Message exchanges

A first step can be to make activity diagrams to define the interaction between system components and the RCC operator. Activity diagrams illustrating the most important actions and functions, as well as the division of responsibility and interactions as shown in in Figs. 6 (normal traffic) and 7 (dense traffic).

A next step can be to make state diagrams to describe how changes between these two modes are performed. A simple state diagram for the autonomous controller is shown in Fig. 8. The diagram contains four states:

1. Idle: The controller waits for a transfer of control from the RCC operator.
2. Automatic navigation: The controller is in charge. If the traffic becomes too dense, a request to intervene (RTI) is issued to the RCC operator.
3. Transfer control: The controller continues controlling the ship while waiting for the operator to take control. If the situation worsens ($T_{\text {DL}}$ expires, see Sect. 3), an MRC is activated.
4. Minimum risk condition (MRC): This could be to stop and hold a position until the operator takes control.

From these descriptions, other UML tools, such as sequence diagrams can be used to detail the communication between the ship the and RCC.

## Conclusion

This paper has described how the operational scenario for an autonomous ship system can be generalized through UML use cases to an operational envelope, consisting of an operational state space ${\mathbb {O}}$ and a functional mapping ***FM***. The paper also shows how the generalized operational envelope can be used to simplify the approval of new operational scenarios and corresponding operational envelopes as long as the new use cases can be proven to be sub-sets of the original. Furthermore, the paper proposes a characterisation of ship autonomy by response time parameters for humans and automation. This does not replace other forms of characterisations, but can provide a more consistent and verifiable way of classifying different degrees of autonomy, which in turn can be used in the design and approval processes. Finally, it suggests how the use of UML can link the approval basis directly to the system design.
